# rMotifGen: random motif generator for DNA and protein sequences

**DOI:** 10.1186/1471-2105-8-292

**Published:** 2007-08-07

**Authors:** Eric C Rouchka, C Timothy Hardin

**Affiliations:** 1 Department of Computer Engineering and Computer Science, University of Louisville, Louisville, KY 40292, USA

## Abstract

**Background:**

Detection of short, subtle conserved motif regions within a set of related DNA or amino acid sequences can lead to discoveries about important regulatory domains such as transcription factor and DNA binding sites as well as conserved protein domains. In order to help assess motif detection algorithms on motifs with varying properties and levels of conservation, we have developed a computational tool, rMotifGen, with the sole purpose of generating a number of random DNA or protein sequences containing short sequence motifs. Each motif consensus can be user-defined, randomly generated, or created from a position-specific scoring matrix (PSSM). Insertions and mutations within these motifs are created according to user-defined parameters and substitution matrices. The resulting sequences can be helpful in mutational simulations and in testing the limits of motif detection algorithms.

**Results:**

Two implementations of rMotifGen have been created, one providing a graphical user interface (GUI) for random motif construction, and the other serving as a command line interface. The second implementation has the added advantages of platform independence and being able to be called in a batch mode. rMotifGen was used to construct sample sets of sequences containing DNA motifs and amino acid motifs that were then tested against the Gibbs sampler and MEME packages.

**Conclusion:**

rMotifGen provides an efficient and convenient method for creating random DNA or amino acid sequences with a variable number of motifs, where the instance of each motif can be incorporated using a position-specific scoring matrix (PSSM) or by creating an instance mutated from its corresponding consensus using an evolutionary model based on substitution matrices. rMotifGen is freely available at: .

## Background

Detection of short, subtle conserved regions within a set of related sequences can be beneficial in determining biologically important regulatory elements such as transcription factor and DNA binding sites, and conserved protein domains. Over 30 software solutions have been published with the underlying goal of detecting subtle, conserved motif sequences within a set of related sequences [1], including implementations using Gibbs sampling routines [2-9], expectation-maximization (EM) [10-12], particle swarm optimization (PSO) [13] and a variety of other methods [12,14-20]. Motif detection algorithms are known to be limited, producing accuracies on the order of 15–25% at the nucleotide level, and 25–35% at the binding site level [1]. Despite the large number of detection algorithms and high levels of inaccuracy, only a limited number of assessments of motif discovery tools have been performed [21,22]. Tompa et al. [22] describe the creation of a DNA benchmarking dataset using the binding sites of known promoter sequences from the TRANSFAC database [23] along with DNA motifs creating using a Markov chain. Creation of this dataset is critically important for the assessment of motif detection approaches. However, the benchmark dataset is limited only to DNA sequences, and those randomly created are done so using a stochastic approach rather than one directed by a mutational model. rMotifGen has been developed as a solution to test various aspects of these limitations by creating simulated DNA and protein sequences where the motifs within the amino acid sequences are allowed to mutate according to a substitution matrix model.

A number of different approaches for generating random biological sequences have previously been presented. The majority of these programs have focused on generating random sequences for the purpose of simulating evolution through mutation events [24-26] or by emitting sequences to resemble those from a particular statistical model. More recently, the program GenRGenS [27] describes a software tool that can expand upon these by incorporating structure into random sequences using context-free grammars and regular expressions.

While each of these approaches is adequate in simulating sequence events on a whole sequence level, a solution has not previously been described to generate completely random sequences that contain regions of similar signals within them. rMotifGen addresses this need by providing a method for randomly creating DNA or amino acid sequences using a simple background model, and then introducing into these sequences subtle motifs. rMotifGen allows for each of the motifs to be incorporated into the individual sequences with different probabilities. In addition, each motif instance is allowed to mutate from the consensus within each sequence using substitution matrix models and PSSMs.

## Implementation

### User input

rMotifGen generates random motifs and sequences based upon two levels of user inputs. The first level requires the user to declare the length, number, and type of sequences (DNA or amino acid), as well as the number of different motifs to create. Based upon the sequence type, the user must provide a background frequency for each residue to create the random sequences. These frequencies are treated as proportionate, which are normalized to one in order to be further considered as percentages. Once the user has entered the first level of overall information, the user is then required to provide the properties for each motif, which can be based on a user-entered consensus, randomly generated sequence, or PSSM. Residue frequencies for each randomly generated consensus must be provided. As a result, the user can provide such information as an amino acid region that is hydrophobic or hydrophilic in nature. Random DNA motifs can be designed to maintain reverse complementary palindromic properties.

Two versions of rMotifGen are provided depending on the needs of the user. The first is a command line interface developed using ANSI g++ that should be portable to any operating system. The second is a GUI developed using Visual Studio^® ^2005 C# that has been tested extensively on Windows^® ^XP. User inputs will be handled differently based upon which version is being used.

### Command line mode

The command line version of rMotifGen version 3.0 is distributed as a gzipped tarball file consisting of four .H header files and four .cpp implementation files. Development of the command line version has proceeded to provide researchers with the ability to use rMotifGen on a variety of computing platforms in an interactive environment and also to allow for creation of input via files so that rMotifGen can be called from batch scripts.

#### Interactive mode

Once the executable for rMotifGen has been created, the interactive mode can be invoked by typing **rMotifGen **at the command line prompt without any parameters. The user is prompted for each of the overall inputs one at a time via the console, followed by the individual parameters for each motif. The resulting random sequences can be output either to a specified file or to the console, depending on the user's desire.

#### Batch mode

Simulations are often very useful to study the behavior patterns of algorithms. This is true as well for motif detection algorithms. Setting up a large number of variable inputs for each of the algorithms may be desired. As a result, the command line version of rMotifGen allows users to read motif parameters from input files that can be automatically generated. This allows rMotifGen to be callable from within a shell script to efficiently design random sequences containing motifs of different composition. The batch mode is invoked with the usage: **rMotifGen [P|N] -f <Filename> **where **P **signifies that the random sequences generated will be protein sequences and **N **signifies they will be amino acid sequences. **Filename **refers to the name of the file where the input data is stored. The input filename is structured using Boulder Data Interchange Format [28] which formats the data in a TAG = VALUE format that can be easily parsed. Two sample input files are provided in the tarball, DNAinput.dat for an example of how to create the file for DNA sequences and AAinput.dat for amino acid sequences. Since the input originates from a file, and the output can be written to a file, the batch mode allows for the possibility of creating a large number of random sequences for simulation purposes.

### GUI mode

**Figure 1:**
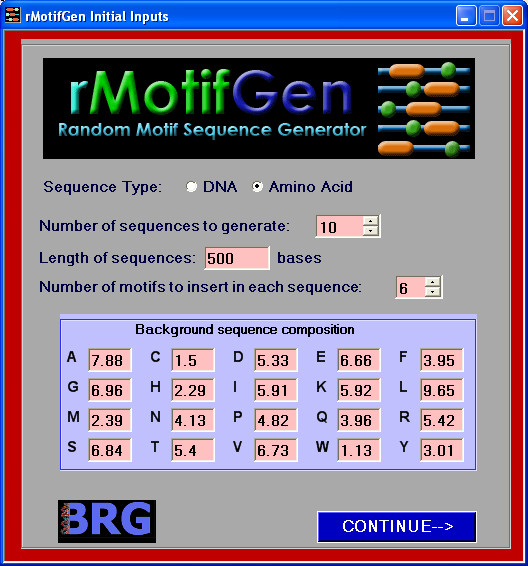
**Initial input screen for rMotifGen**. This screen illustrates an example where the user will be generating amino acid sequences. The default background frequencies presented to the user are based upon the observed residue frequencies in the SWISS-PROT database release 52.0 [29].

**Figure 2:**
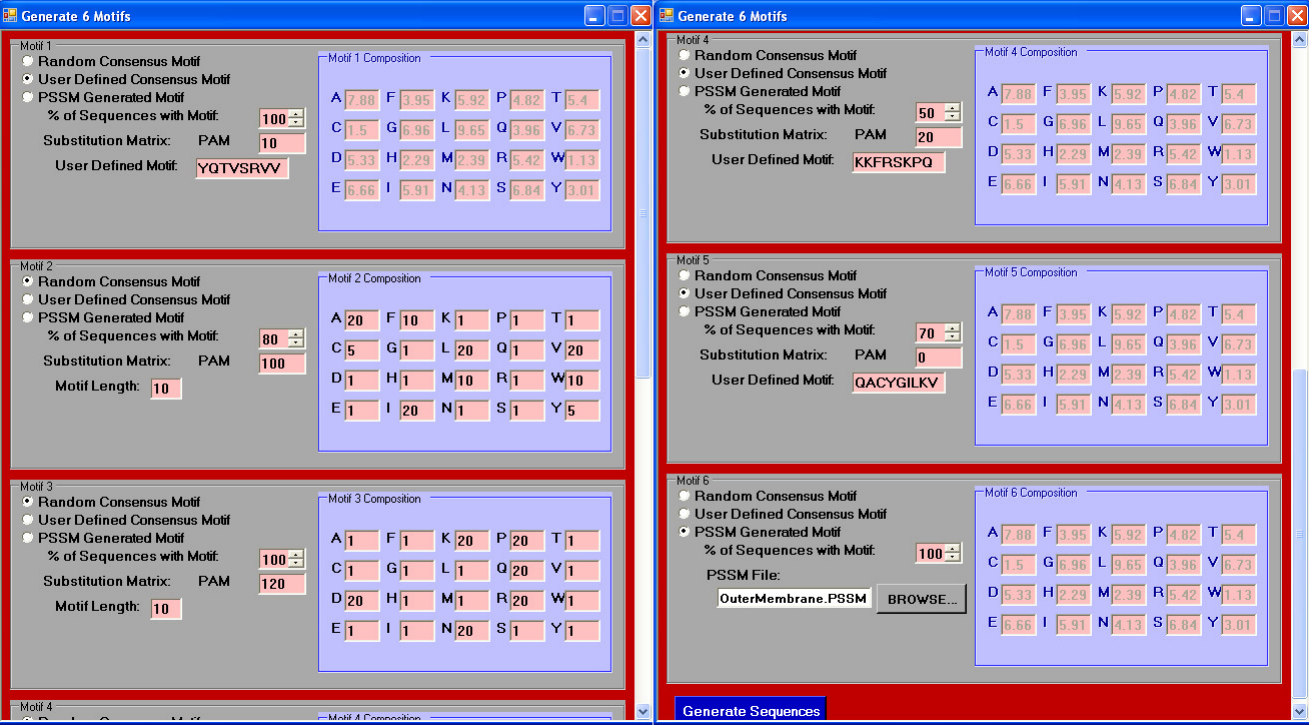
**Motif description page**. In this screen, the user will be allowed to specify the parameters for each motif, including a description of whether it is random or user-defined, what sort of conservation each instance will have to the consensus, what percentage of sequences will have the motif, and the background composition (if the motif is randomly generated).

**Figure 3:**
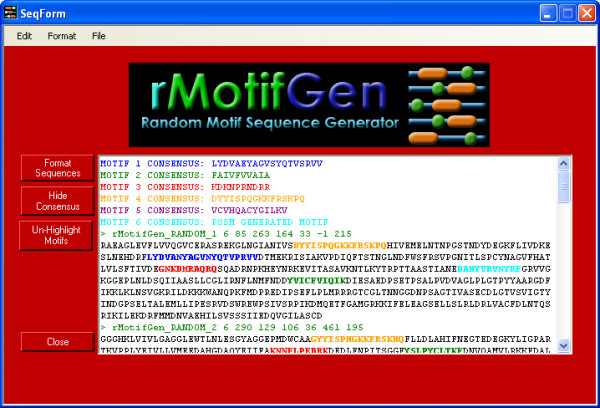
**Resulting random sequences**. This screen shows the consensus motifs and the resulting random sequences. Each of the motif instances can be highlighted and the sequences can be copied or saved to a file.

An initial screen prompting the user for overall sequence properties first pops up when run in GUI mode (Figure 1). The user must provide the initial background frequencies for the desired sequence type. Defaults for DNA sequences are 25% for each residue. For amino acid sequences, the background frequency of each amino acid is set according to the observed frequencies within the SWISS-PROT database [29] release 52.0. When the user presses the "CONTINUE" button, s/he is prompted to enter in the details for each motif consensus to be generated (Figure 2). In this screen, a decision can be made as to whether the consensus is created based on a user-defined sequence, position specific scoring matrix, or if it will be created randomly. In the case that it that it is randomly generated, the user will provide the residue frequencies for motif generation. This allows motifs to be constructed based on a number of different properties. If the motif is a DNA sequence, it can additionally be constrained to be a palindrome. Each randomly generated sequence can have zero or one occurrence of the motif, which is incorporated by the specified percentage of sequences in which it is contained. Two or more occurrences of the same motif into each sequence can be incorporated by adding duplicate records for the same motif. The level of conservation between the consensus motif and each randomly generated instance is provided. In the case where the consensus is not defined by a PSSM, DNA sequences use a straight-forward percent conservation of the consensus to determine the sequence for each motif occurrence, while amino acid sequences are generated from the consensus based on the provided PAM substitution matrix model [30]. The larger the number is for the PAM, the more divergent the motifs will be. A PAM of 0 can be used if complete conservation is desired. When the motif parameters are set, the user presses the "Generate Sequences" button which creates the sequences and presents them in a formatted output screen (Figure 3) where they can be copied to the clipboard or saved to a file.

### Random sequence generation

For both the GUI and command line interfaces, the sequences are randomly generated based upon the background residue frequencies. In order to make rMotifGen more robust, the frequencies entered are not required to sum to either 1 or 100. Rather, they are treated as ratios which are subsequently normalized. Each position within the sequence is treated as a completely separate entity. For the command line interface, the ANSI standard drand48 family of pseudo-random number generators is used.

After the sequences have been randomly generated, consensus motifs are created. These can either be set according to the user-defined consensus pattern or randomly generated based upon the motif residue frequencies. Each random sequence is then considered to determine whether or not it will contain an instance of the current motif. If the motif is selected for inclusion, the background sequence is examined to see which sites are available for the motif position such that two motifs are not overlapping in order to prevent truncated motifs. The current version of rMotifGen does not allow for overlapping motifs, but does allow for them to be randomly ordered. The motif instance is then created using the model of conservation provided, via a PAM lookup table, PSSM, or by a percent conservation variable.

### Output

Each random sequence is presented in FASTA file format. The descriptor line begins with a '>' followed by an identifier rMotifGen_RandSeq_<seqnum> which uniquely identifies the sequence. The remainder of the header contains the number of motifs created, followed by each individual beginning location. If a motif is omitted, its positional value is -1. The remainder of the FASTA file is the actual sequence data. Figure 3 illustrates an example FASTA sequence.

## Results

A sample amino acid sequence set of ten sequences of length 500 residues with six allowed motifs per sequence was generated from rMotifGen. Background residue frequencies for the data set was taken from the observed amino acid frequencies in SWISS-PROT [29] release 52.0 dated 06-Mar-07 [28,31]. The first motif consensus, LYDVAEYAGVSYQTVSRVV, was entered in as a helix-turn-helix (HTH) motif from *E. coli *lactose operon repressor Lac1 [32]. The second and third consensuses were randomly generated, where the motif residue frequencies were heavily weighted towards hydrophobic and hydrophilic amino acids, respectively. The fourth motif consensus, DVYYISPQGKKFRSKPQ, represents a partial region from the methyl CpG binding domain (MBD) extracted from PROSITE [33] entry PDOC50982. The fifth consensus, VCVHQACYGILKV, was taken from the multiple alignment for the plant homeodomain (PHD) type zinc finger (PROSITE entry PDOC50016). The final motif was generated from a PSSM for the outer membrane motif described in Neuwald, et al. [3]. PAM matrices used for each of these sequences were 10, 100, 200, 20, and 0 respectively, indicating that the second and third motifs are more likely to incorporate mutations, and the fifth motif is 100% conserved from the consensus sequence. Note that since the sixth motif is PSSM-generated, it does not have an associated PAM matrix. Conservation of the motifs within each sequence was set to 100%, 80%, 100%, 50%, 70%, and 75% respectively, indicating the likelihood that each sequence contains each of the six motifs. The resulting motifs generated are listed in Table 1, and their locations are listed in Table 2. Complete sequences are available [see Additional file 1].

**Table 1:** Motifs for randomly generated sequences

**Seq. #**	**Motif 1**	**Motif 2**	**Motif 3**	**Motif 4**	**Motif 5**	**Motif 6**
CONS.	LYDVAEYAGVSYQTVSRVV	FAIVFVVAIA	KDKNPRNDRR	DYYISPQGKKFRSKPQ	VCVHQACYGILKV	PSSM

1	LYDVANYAGVNYQTVPRVV	YVICFVIQIK	GNKDMRAQRQ	DYYISPQGKKFRSKPQ	--	DAHYVRVNYRF
2	LYDVAEYAGVSYQTVSRTV	YSLPYCLTKF	KNNFLPEDRK	GYYISPHGKKFRSKHQ	VCVHQACYGILKV	KTFYLGAGYRY
3	LYDVAEYAGVSYQTVSRVV	YALGGLIESA	NDRSNRGKPW	---	VCVHQACYGILKV	KAVYAGLGVKF
4	LYDVADYAGVSYQAVSRVV	FDAGFVLPAT	HGTKSDKTIR	---	VCVHQACYGILKV	DQVTLGAGMDF
5	LYNVAEYIGVSYNTVSRVV	AQIVVCLAGG	KEKIPKEVRK	---	---	DQYHASAGYKF
6	LYDVAEYAGVSYQTVSRVV	FGIVYVLANA	KDEDLRNSRR	DFYISAQGKKFRSKPQ	VCVHQACYGILKV	RNWYVRAGYDY
7	LYDVAEYAGVVYQTVSKVV	PSMLLFVEIA	KDKNNPNQSS	--	VCVHQACYGILKV	NAVYIGLGVRY
8	LYDVAEYAGVSYQTVSRVV	FFVVFSVVIT	RQKNAEHDRR	--	VCVHQACYGILKV	KTYHVGLGFDY
9	LYDVAEYNGISYEVVSRVV	DAIIFANNID	MEKNLWDERM	DYYISPQGKKFRVNPN	VCVHQACYGILKV	DAYYARAGVDF
10	LYDIAEYAGVSYQTVSRVV	FAMVIGVSIG	GKKEPRYEQP	DYYIWPKGKKFKSKPQ	VCVHQACYGILKV	PNYHAGLGLRY

**Table 2:** Actual and predicted motif begin locations

**SEQ. NUMBER**	**Motif 1**			**Motif 2**			**Motif 3**			**Motif 4**			**Motif 5**			**Motif 6**		
	**R**	**M**	**G**	**R**	**M**	**G**	**R**	**M**	**G**	**R**	**M**	**G**	**R**	**M**	**G**	**R**	**M**	**G**

1	85	85	85	263	--	--	164	--	--	33	33	33	--	--	--	215	215	--
2	290	290	290	129	--	--	106	--	--	36	36	36	461	461	***464***	195	195	--
3	332	332	332	286	--	--	203	--	--	--	--	--	354	354	***357***	93	93	--
4	20	20	20	112	--	--	74	--	--	--	--	--	378	378	***381***	239	239	--
5	150	150	150	26	--	--	133	--	--	--	--	--	--	--	--	170	170	--
6	187	187	187	17	--	--	455	--	--	247	247	247	473	473	***476***	413	***291***	--
7	334	334	334	461	--	--	488	--	--	--	--	--	394	394	***397***	80	80	--
8	259	259	259	396	--	--	163	--	--	--	--	--	70	70	***73***	445	445	--
9	197	197	197	330	--	--	480	--	--	443	443	443	314	314	***317***	397	397	--
10	359	359	359	51	--	--	337	--	--	108	108	108	31	31	***34***	141	141	--

**R**: rMotifGen randomly assigned motifs; **M**: MEME assigned motifs; **G**: Gibbs Sampler assigned motifs. Each of the start positions have been modified so that each sequence begins at 0, to comply with rMotifGen. Sites incorrectly found are listed in a bold, italic font. This includes the motif 5 positions found by the Gibbs Sampler which are offset by three bases

rMotifGen can be used to create benchmark data sets for motif detection algorithms. In order to demonstrate this capability, the test set described above was used as input into the MEME web server [34] and the Gibbs motif sampler [35]. Results for MEME and Gibbs sampler are found in Table 2.

MEME had the parameters set for zero or one occurrence per sequence, and a total of six motifs to detect. MEME was able to locate the exact positions for each occurrence of motif one, four, and five while finding all but one occurrence of motif six (Table 2). However, MEME was unable to locate motifs two and three in any part. Since MEME was forced to find a total of six motifs, two additional patterns were found where each pattern had two occurrences (results not shown) [see Additional file 2].

For the Gibbs sampler, the number of motif patterns was set to six with motif widths of 10, 10, 12, 17, 19, and 11. The estimated site count for each motif type was set to 10. The remaining parameters were left at the default values. As Table 2 indicates, the Gibbs sampler was not as effective at locating the motif sites as MEME. Gibbs found the exact locations for the motifs one and four, while find the locations with a three base offset for motif five. Gibbs was unable to find the occurrences for the motif patterns two, three and six. An additional three motif patterns were found (results not shown – see supplementary materials) which do not correspond to either the MEME patterns or the expected motif patterns. Results for the Gibbs sampler are provided [see Additional file 3].

## Discussion

rMotifGen provides an effective method for constructing motifs in DNA and amino acid sequences according to substitution matrices and PSSMs which have biological relevance. One of the limitations to the current approach, however, is that the background residues are randomly chosen using a Bernoulli methodology. The implementation generates the random background sequences such that each of the sequences generated has the same residue distribution. A more biologically relevant approach would be to assign each individual background sequence a distribution which takes into account a higher order sequence organization. A model (such as a hidden Markov model approach) which takes into account factors such as dinucleotide and hexamer frequencies for DNA sequences would provide sequences more likely to occur in nature. rMotifGen does not incorporate these higher order methodologies for the background sequences at this point in time in order to maintain a reasonable number of parameters (each background sequence could potentially belong to a unique organism with a different underlying model), and since doing so may help create sequences that are in effect trained for a particular motif detection approach. Future releases will provide for more intelligent background sequence modeling.

## Conclusion

Detection of short, subtle motif signals within DNA and amino acid sequences remains a difficult problem due to the fact that biological signals may not be highly conserved. Testing of motif detection approaches using benchmark standards is important. Our solution, rMotifGen, demonstrates that creation of random motif datasets under certain evolutionary constraints can be used to determine the limitations of motif detection algorithms. By providing both GUI and command line versions, rMotifGen should be flexible to suit any desired need, including the construction of large scale sets for simulations.

## Availability and requirements

rMotifGen can be accessed at: . Two versions, one providing a graphical interactive environment, and the other serving as a more portable command line interface, are available. In addition, an html form-based interface into the command line version is available at the above website.

The interactive version was developed using C# within VisualStudio^® ^2005 on a Windows^® ^XP system. The GUI executable is freely available to all users. Microsoft's^® ^.NET Framework Version 2.0 Redistribution Package (×86) is required, and can be freely downloaded from . It has been tested on a Windows^® ^XP Professional system, but should also run on any Windows^® ^system using Windows^® ^98 or later.

Distribution of the command line interface is accomplished under the GNU public license for all users. It was developed using ANSI C++, and should be portable to any system with an ANSI C++ compiler, such as gnu's g++. It has been fully tested using g++ 4.0.2 on a system using SUSE^® ^Linux 10.0.

## Abbreviations

American National Standards Institute (ANSI), Bioinformatics Research Group (BRG), expectation-maximization (EM), graphical user interface (GUI), helix-turn-helix (HTH), methyl CpG binding domain (MBD), multiple expectation-maximization for motif elicitation (MEME), plant homeodomain (PHD), point accepted mutation (PAM), particle swarm optimization (PSO), position specific scoring matrix (PSSM)

## Authors' contributions

ECR and CTH worked together in the design and testing phase of software development. ECR is the main author of the code. Both authors have read and approved this manuscript.

## Supplementary Material

Additional file 1 rMotifGen-SAMPLE_AA.fa. Example randomly generated amino acid sequence file used in the manuscript. Click here for file

Additional file 2 Meme-21932.results.html. Resulting motif detection for the random sequences using MEME. Click here for file

Additional file 3 gibbsSampler.results. Resulting motif detection for the random sequences using the Gibbs Sampler. Click here for file
