# Supplementary material for: rMotifGen: random motif generator for DNA and protein sequences
Source: BMC Bioinformatics. 2007 Aug 7;8:292. doi: 10.1186/1471-2105-8-292 (PMC1963340; doi:10.1186/1471-2105-8-292)
Supplement: Additional file 2 — Meme-21932.results.html. Resulting motif detection for the random sequences using MEME. [file 1471-2105-8-292-S2.html]

 
 
 
 
 MEME 
 
  TD.invisible { color: '#D5F0FF'; }
  TD.c0 { background: aqua; color: black; }
  TD.cw0 { background: aqua; color: black; font: 50% sans-serif; }
  TD.c1 { background: blue; color: white; }
  TD.cw1 { background: blue; color: white; font: 50% sans-serif; }
  TD.c2 { background: red; color: white; }
  TD.cw2 { background: red; color: white; font: 50% sans-serif; }
  TD.c3 { background: fuchsia; color: black; }
  TD.cw3 { background: fuchsia; color: black; font: 50% sans-serif; }
  TD.c4 { background: yellow; color: black; }
  TD.cw4 { background: yellow; color: black; font: 50% sans-serif; }
  TD.c5 { background: lime; color: black; }
  TD.cw5 { background: lime; color: black; font: 50% sans-serif; }
  TD.c6 { background: teal; color: white; }
  TD.cw6 { background: teal; color: white; font: 50% sans-serif; }
  TD.c7 { background: #444444; color: white; }
  TD.cw7 { background: #444444; color: white; font: 50% sans-serif; }
  TD.c8 { background: green; color: white; }
  TD.cw8 { background: green; color: white; font: 50% sans-serif; }
  TD.c9 { background: silver; color: black; }
  TD.cw9 { background: silver; color: black; font: 50% sans-serif; }
  TD.c10 { background: purple; color: white; }
  TD.cw10 { background: purple; color: white; font: 50% sans-serif; }
  TD.c11 { background: olive; color: black; }
  TD.cw11 { background: olive; color: black; font: 50% sans-serif; }
  TD.c12 { background: navy; color: white; }
  TD.cw12 { background: navy; color: white; font: 50% sans-serif; }
  TD.c13 { background: maroon; color: white; }
  TD.cw13 { background: maroon; color: white; font: 50% sans-serif; }
  TD.c14 { background: black; color: white; }
  TD.cw14 { background: black; color: white; font: 50% sans-serif; }
  TD.c15 { background: white; color: black; }
  TD.cw15 { background: white; color: black; font: 50% sans-serif; }
  B.red { color: red; }
  TD.red { color: red; }
  TH.red { color: red; }
  B.blue { color: blue; }
  TD.blue { color: blue; }
  TH.blue { color: blue; }
  B.orange { color: orange; }
  TD.orange { color: orange; }
  TH.orange { color: orange; }
  B.green { color: green; }
  TD.green { color: green; }
  TH.green { color: green; }
  B.black { color: black; }
  TD.black { color: black; }
  TH.black { color: black; }
  B.magenta { color: magenta; }
  TD.magenta { color: magenta; }
  TH.magenta { color: magenta; }
  B.pink { color: pink; }
  TD.pink { color: pink; }
  TH.pink { color: pink; }
  B.yellow { color: yellow; }
  TD.yellow { color: yellow; }
  TH.yellow { color: yellow; }
  B.turquoise { color: turquoise; }
  TD.turquoise { color: turquoise; }
  TH.turquoise { color: turquoise; }
 
 
 
   
  
     Command line  
     Training Set  
     First Motif  
     Summary of Motifs  
     Termination  
     Explanation   
 

 
 
   Search sequence databases with these motifs using  MAST. 
   Submit these motifs to  BLOCKS multiple alignment processor. 
   Build and use a motif-based hidden Markov model (HMM) using  Meta-MEME. 
  
    
      MEME - Motif discovery tool  
    
 
MEME version 3.5.4 (Release date: 3.5.4)
 
For further information on how to interpret these results or to get
a copy of the MEME software please access  http://meme.nbcr.net. 
 
This file may be used as input to the MAST algorithm for searching
sequence databases for matches to groups of motifs.  MAST is available
for interactive use and downloading at  http://meme.nbcr.net. 

    
      REFERENCE  
    
 
If you use this program in your research, please cite:
 
Timothy L. Bailey and Charles Elkan,
"Fitting a mixture model by expectation maximization to discover
motifs in biopolymers", Proceedings of the Second International
Conference on Intelligent Systems for Molecular Biology, pp. 28-36,
AAAI Press, Menlo Park, California, 1994.

    
      TRAINING SET  
    
 
DATAFILE= C:\HOME\2007_FILES\Documents\rMOTIFGEN\AASequences.fa
ALPHABET= ACDEFGHIKLMNPQRSTVWY
Sequence name            Weight Length  Sequence name            Weight Length  
-------------            ------ ------  -------------            ------ ------  
rMotifGen_RANDOM_1       1.0000    500  rMotifGen_RANDOM_2       1.0000    500  
rMotifGen_RANDOM_3       1.0000    500  rMotifGen_RANDOM_4       1.0000    500  
rMotifGen_RANDOM_5       1.0000    500  rMotifGen_RANDOM_6       1.0000    500  
rMotifGen_RANDOM_7       1.0000    500  rMotifGen_RANDOM_8       1.0000    500  
rMotifGen_RANDOM_9       1.0000    500  rMotifGen_RANDOM_10      1.0000    500  
 
 
 
 

    
      COMMAND LINE SUMMARY  
    
 
This information can also be useful in the event you wish to report a
problem with the MEME software.

command: meme /home/meme/meme354/LOGS/meme.21932.data -protein -mod zoops -nmotifs 6 -maxsites 10 -minw 6 -maxw 50 -evt 1e100 -time 7200 -maxsize 60000 -nostatus -maxiter 20 -dir /home/meme/meme354 

model:  mod=         zoops    nmotifs=         6    evt=        1e+100
object function=  E-value of product of p-values
width:  minw=            6    maxw=           50    minic=        0.00
width:  wg=             11    ws=              1    endgaps=       yes
nsites: minsites=        2    maxsites=       10    wnsites=       0.8
theta:  prob=            1    spmap=         pam    spfuzz=        120
em:     prior=       megap    b=           25000    maxiter=        20
        distance=    1e-05
data:   n=            5000    N=              10

sample: seed=            0    seqfrac=         1
Dirichlet mixture priors file: prior30.plib
Letter frequencies in dataset:
A 0.077 C 0.017 D 0.054 E 0.061 F 0.039 G 0.072 H 0.022 I 0.058 K 0.060 
L 0.095 M 0.021 N 0.037 P 0.047 Q 0.040 R 0.058 S 0.062 T 0.052 V 0.077 
W 0.011 Y 0.040 
Background letter frequencies (from dataset with add-one prior applied):
A 0.077 C 0.017 D 0.054 E 0.061 F 0.039 G 0.072 H 0.022 I 0.058 K 0.060 
L 0.095 M 0.021 N 0.037 P 0.047 Q 0.040 R 0.058 S 0.062 T 0.052 V 0.077 
W 0.011 Y 0.040 
 
 

    
   
     P  
     N        MOTIF  1       width = 19         sites =  10        llr = 480        E-value = 4.6e-099 
    
 
    Simplified  A  : : : : a : : 8 : : : : : 1 : : : : :
    pos.-specific  C  : : : : : : : : : : : : : : : : : : :
    probability  D  : : 9 : : 1 : : : : : : : : : : : : :
    matrix  E  : : : : : 8 : : : : : : 1 : : : : : :
    F  : : : : : : : : : : : : : : : : : : :
    G  : : : : : : : : a : : : : : : : : : :
    H  : : : : : : : : : : : : : : : : : : :
    I  : : : 1 : : : 1 : 1 : : : : : : : : :
    K  : : : : : : : : : : : : : : : : 1 : :
    L  a : : : : : : : : : : : : : : : : : :
    M  : : : : : : : : : : : : : : : : : : :
    N  : : 1 : : 1 : 1 : : 1 : 1 : : : : : :
    P  : : : : : : : : : : : : : : : 1 : : :
    Q  : : : : : : : : : : : : 8 : : : : : :
    R  : : : : : : : : : : : : : : : : 9 : :
    S  : : : : : : : : : : 8 : : : : 9 : : :
    T  : : : : : : : : : : : : : 8 : : : 1 :
    V  : : : 9 : : : : : 9 1 : : 1 a : : 9 a
    W  : : : : : : : : : : : : : : : : : : :
    Y  : a : : : : a : : : : a : : : : : : :
  . 
               bits      6.5                    
                      5.8                    
                      5.2                    
                      4.5                       
      Information   3.9                               
      content   3.2                                       
    (69.2 bits) 2.6                                       
                      1.9                                       
                      1.3                                       
                      0.6                                       
                      0.0    
  . 
    Multilevel                 L      Y      D      V      A      E      Y      A      G      V      S      Y      Q      T      V      S      R      V      V   
    consensus                                                     
    sequence                                                      
                                                                
                                                                

  . 
  NAME       START   P-VALUE           SITES   
  rMotifGen_RANDOM_8    260  1.10e-23 
    DKAPGLQPVA     L      Y      D      V      A      E      Y      A      G      V      S      Y      Q      T      V      S      R      V      V       IIQFESCIYI 
  rMotifGen_RANDOM_6    188  1.10e-23 
    LFTVAMDSEI     L      Y      D      V      A      E      Y      A      G      V      S      Y      Q      T      V      S      R      V      V       LGTAKVGRSL 
  rMotifGen_RANDOM_3    333  1.10e-23 
    VGGNYGRLML     L      Y      D      V      A      E      Y      A      G      V      S      Y      Q      T      V      S      R      V      V       RLIVCVHQAC 
  rMotifGen_RANDOM_10    360  2.76e-23 
    SDEFGVTFGE     L      Y      D      I      A      E      Y      A      G      V      S      Y      Q      T      V      S      R      V      V       RELDASGNTK 
  rMotifGen_RANDOM_2    291  7.28e-23 
    EFWIKWRDGW     L      Y      D      V      A      E      Y      A      G      V      S      Y      Q      T      V      S      R      T      V       DAMKTSQLAI 
  rMotifGen_RANDOM_7    335  5.20e-21 
    ATFNPKPRRI     L      Y      D      V      A      E      Y      A      G      V      V      Y      Q      T      V      S      K      V      V       RQFKDSNHLS 
  rMotifGen_RANDOM_4    21  5.97e-21 
    TVLALRIQPP     L      Y      D      V      A      D      Y      A      G      V      S      Y      Q      A      V      S      R      V      V       LIGKYKIKWK 
  rMotifGen_RANDOM_1    86  5.07e-20 
    KESLNEHDRF     L      Y      D      V      A      N      Y      A      G      V      N      Y      Q      T      V      P      R      V      V       DTMEKRISIA 
  rMotifGen_RANDOM_5    151  1.09e-19 
    VRKSNPEEPF     L      Y      N      V      A      E      Y      I      G      V      S      Y      N      T      V      S      R      V      V       VDQYHASAGY 
  rMotifGen_RANDOM_9    198  4.27e-19 
    VSKRVDVKIT     L      Y      D      V      A      E      Y      N      G      I      S      Y      E      V      V      S      R      V      V       QLHVKLFRGQ 
 
    
      	Motif 1 block diagrams  
    
 
  Name Lowest p-value    Motifs
 
     rMotifGen_RANDOM_8
  1.1e-23
    
    
   1
    
   
  
 
     rMotifGen_RANDOM_6
  1.1e-23
    
    
   1
    
   
  
 
     rMotifGen_RANDOM_3
  1.1e-23
    
    
   1
    
   
  
 
     rMotifGen_RANDOM_10
  2.8e-23
    
    
   1
    
   
  
 
     rMotifGen_RANDOM_2
  7.3e-23
    
    
   1
    
   
  
 
     rMotifGen_RANDOM_7
  5.2e-21
    
    
   1
    
   
  
 
     rMotifGen_RANDOM_4
  6e-21
    
    
   1
    
   
  
 
     rMotifGen_RANDOM_1
  5.1e-20
    
    
   1
    
   
  
 
     rMotifGen_RANDOM_5
  1.1e-19
    
    
   1
    
   
  
 
     rMotifGen_RANDOM_9
  4.3e-19
    
    
   1
    
   
  
  SCALE
     
     | 
     | 
     | 
     | 
     | 
     | 
     | 
     | 
     | 
     | 
     | 
     | 
     | 
     | 
     | 
     | 
     | 
     | 
     | 
     | 
    1 
     25 
     50 
     75 
     100 
     125 
     150 
     175 
     200 
     225 
     250 
     275 
     300 
     325 
     350 
     375 
     400 
     425 
     450 
     475 
   
 

    
      	Motif 1 in BLOCKS format  
    
 
 
 
 
  
 to  BLOCKS multiple alignment processor.  
    
      	Motif 1 position-specific scoring matrix  
    
 
 

    
      	Motif 1 position-specific probability matrix  
    
 
 

    
      	Motif 1 regular expression  
    
LYDVAEYAGVSYQTVSRVV

 


Time  3.94 secs.

 

    
   
     P  
     N        MOTIF  2       width = 13         sites =   8        llr = 315        E-value = 9.6e-071 
    
 
    Simplified  A  : : : : : a : : : : : : :
    pos.-specific  C  : a : : : : a : : : : : :
    probability  D  : : : : : : : : : : : : :
    matrix  E  : : : : : : : : : : : : :
    F  : : : : : : : : : : : : :
    G  : : : : : : : : a : : : :
    H  : : : a : : : : : : : : :
    I  : : : : : : : : : a : : :
    K  : : : : : : : : : : : a :
    L  : : : : : : : : : : a : :
    M  : : : : : : : : : : : : :
    N  : : : : : : : : : : : : :
    P  : : : : : : : : : : : : :
    Q  : : : : a : : : : : : : :
    R  : : : : : : : : : : : : :
    S  : : : : : : : : : : : : :
    T  : : : : : : : : : : : : :
    V  a : a : : : : : : : : : a
    W  : : : : : : : : : : : : :
    Y  : : : : : : : a : : : : :
  . 
               bits      6.5              
                      5.8                 
                      5.2                 
                      4.5                   
      Information   3.9                          
      content   3.2                           
    (56.8 bits) 2.6                           
                      1.9                           
                      1.3                           
                      0.6                           
                      0.0    
  . 
    Multilevel                 V      C      V      H      Q      A      C      Y      G      I      L      K      V   
    consensus                                         
    sequence                                          
                                                    
                                                    

  . 
  NAME       START   P-VALUE           SITES   
  rMotifGen_RANDOM_10    32  8.18e-18 
    HTEYPQTDFA     V      C      V      H      Q      A      C      Y      G      I      L      K      V       LIASSVRFAM 
  rMotifGen_RANDOM_9    315  8.18e-18 
    SGIVEQKGPP     V      C      V      H      Q      A      C      Y      G      I      L      K      V       FVQDAIIFAN 
  rMotifGen_RANDOM_8    71  8.18e-18 
    TGVRAPTTLA     V      C      V      H      Q      A      C      Y      G      I      L      K      V       PKITEYVLLQ 
  rMotifGen_RANDOM_7    395  8.18e-18 
    TPIAPQVRNG     V      C      V      H      Q      A      C      Y      G      I      L      K      V       QRQRIGWICI 
  rMotifGen_RANDOM_6    474  8.18e-18 
    RRQEEAIILT     V      C      V      H      Q      A      C      Y      G      I      L      K      V       ICHTRGPTLP 
  rMotifGen_RANDOM_4    379  8.18e-18 
    FSALKQGRRV     V      C      V      H      Q      A      C      Y      G      I      L      K      V       RSGRRKGALF 
  rMotifGen_RANDOM_3    355  8.18e-18 
    QTVSRVVRLI     V      C      V      H      Q      A      C      Y      G      I      L      K      V       PKSHLVTTDV 
  rMotifGen_RANDOM_2    462  8.18e-18 
    PLDANPLKNR     V      C      V      H      Q      A      C      Y      G      I      L      K      V       DPHCKYRLAR 
 
    
      	Motif 2 block diagrams  
    
 
  Name Lowest p-value    Motifs
 
     rMotifGen_RANDOM_10
  8.2e-18
    
    
   2
    
   
  
 
     rMotifGen_RANDOM_9
  8.2e-18
    
    
   2
    
   
  
 
     rMotifGen_RANDOM_8
  8.2e-18
    
    
   2
    
   
  
 
     rMotifGen_RANDOM_7
  8.2e-18
    
    
   2
    
   
  
 
     rMotifGen_RANDOM_6
  8.2e-18
    
    
   2
    
   
  
 
     rMotifGen_RANDOM_4
  8.2e-18
    
    
   2
    
   
  
 
     rMotifGen_RANDOM_3
  8.2e-18
    
    
   2
    
   
  
 
     rMotifGen_RANDOM_2
  8.2e-18
    
    
   2
    
   
  
  SCALE
     
     | 
     | 
     | 
     | 
     | 
     | 
     | 
     | 
     | 
     | 
     | 
     | 
     | 
     | 
     | 
     | 
     | 
     | 
     | 
     | 
    1 
     25 
     50 
     75 
     100 
     125 
     150 
     175 
     200 
     225 
     250 
     275 
     300 
     325 
     350 
     375 
     400 
     425 
     450 
     475 
   
 

    
      	Motif 2 in BLOCKS format  
    
 
 
 
 
  
 to  BLOCKS multiple alignment processor.  
    
      	Motif 2 position-specific scoring matrix  
    
 
 

    
      	Motif 2 position-specific probability matrix  
    
 
 

    
      	Motif 2 regular expression  
    
VCVHQACYGILKV

 


Time  7.74 secs.

 

    
   
     P  
     N        MOTIF  3       width = 17         sites =   5        llr = 222        E-value = 4.0e-026 
    
 
    Simplified  A  : : : : : 2 : : : : : : : : : : :
    pos.-specific  C  : : : : : : : : : : : : : : : : 2
    probability  D  8 : : : : : : : : : : : : : : : :
    matrix  E  : : : : : : : : : : : : : : : : 2
    F  : 2 : : : : : : : : a : : : : : 2
    G  2 : : : : : : a : : : : : : : : :
    H  : : : : : : 2 : : : : : : : 2 : 2
    I  : : : a : : : : : : : : : : : : :
    K  : : : : : : 2 : a a : 2 : 8 : : :
    L  : : : : : : : : : : : : : : : : :
    M  : : : : : : : : : : : : : : : : :
    N  : : : : : : : : : : : : : 2 : 2 2
    P  : : : : : 8 : : : : : : : : 8 : :
    Q  : : : : : : 6 : : : : : : : : 8 :
    R  : : : : : : : : : : : 8 : : : : :
    S  : : : : 8 : : : : : : : 8 : : : :
    T  : : : : : : : : : : : : : : : : :
    V  : : : : : : : : : : : : 2 : : : :
    W  : : : : 2 : : : : : : : : : : : :
    Y  : 8 a : : : : : : : : : : : : : :
  . 
               bits      6.5                  
                      5.8                  
                      5.2                  
                      4.5                    
      Information   3.9                             
      content   3.2                                  
    (63.9 bits) 2.6                                   
                      1.9                                   
                      1.3                                   
                      0.6                                   
                      0.0    
  . 
    Multilevel                 D      Y      Y      I      S      P      Q      G      K      K      F      R      S      K      P      Q      C   
    consensus                 G    F        W    A    H            K    V    N    H    N    E  
    sequence                              K                      F  
                                                            H  
                                                            N  

  . 
  NAME       START   P-VALUE           SITES   
  rMotifGen_RANDOM_1    34  5.19e-23 
    GLNGIANIVS     D      Y      Y      I      S      P      Q      G      K      K      F      R      S      K      P      Q      H       IVEMELNTNP 
  rMotifGen_RANDOM_6    248  7.68e-20 
    AIIPDRANKR     D      F      Y      I      S      A      Q      G      K      K      F      R      S      K      P      Q      E       GNSLLISQAV 
  rMotifGen_RANDOM_10    109  4.32e-19 
    MEVHFAYSTE     D      Y      Y      I      W      P      K      G      K      K      F      K      S      K      P      Q      C       MTLKLPTLLP 
  rMotifGen_RANDOM_2    37  1.84e-18 
    GGEPMDWCAA     G      Y      Y      I      S      P      H      G      K      K      F      R      S      K      H      Q      F       LLDLAHIFNE 
  rMotifGen_RANDOM_9    444  4.46e-18 
    GILLEHYFNV     D      Y      Y      I      S      P      Q      G      K      K      F      R      V      N      P      N      N       ADSKHMVALF 
 
    
      	Motif 3 block diagrams  
    
 
  Name Lowest p-value    Motifs
 
     rMotifGen_RANDOM_1
  5.2e-23
    
    
   3
    
   
  
 
     rMotifGen_RANDOM_6
  7.7e-20
    
    
   3
    
   
  
 
     rMotifGen_RANDOM_10
  4.3e-19
    
    
   3
    
   
  
 
     rMotifGen_RANDOM_2
  1.8e-18
    
    
   3
    
   
  
 
     rMotifGen_RANDOM_9
  4.5e-18
    
    
   3
    
   
  
  SCALE
     
     | 
     | 
     | 
     | 
     | 
     | 
     | 
     | 
     | 
     | 
     | 
     | 
     | 
     | 
     | 
     | 
     | 
     | 
     | 
     | 
    1 
     25 
     50 
     75 
     100 
     125 
     150 
     175 
     200 
     225 
     250 
     275 
     300 
     325 
     350 
     375 
     400 
     425 
     450 
     475 
   
 

    
      	Motif 3 in BLOCKS format  
    
 
 
 
 
  
 to  BLOCKS multiple alignment processor.  
    
      	Motif 3 position-specific scoring matrix  
    
 
 

    
      	Motif 3 position-specific probability matrix  
    
 
 

    
      	Motif 3 regular expression  
    
[DG][YF]YI[SW][PA][QHK]GKKF[RK][SV][KN][PH][QN][CEFHN]

 


Time 11.94 secs.

 

    
   
     P  
     N        MOTIF  4       width = 11         sites =  10        llr = 196        E-value = 1.0e-004 
    
 
    Simplified  A  : 4 : : 4 : 4 : : : :
    pos.-specific  C  : : : : : : : : : : :
    probability  D  4 : : : : : : : : 3 :
    matrix  E  : : : : : : : : : : :
    F  : : 1 : 1 : : : 1 : 6
    G  : : : : : 7 : 8 : : :
    H  : : 1 3 : : : : : : :
    I  : : : : 1 : : : : : :
    K  3 : : : : : : : : 2 :
    L  : : : : 2 : 4 : 1 : :
    M  : : : : : : 1 : 1 : :
    N  1 1 : : : : : 1 : 1 :
    P  1 1 : : : : : : : : :
    Q  : 2 : : : : : : : : :
    R  1 : : : : 2 : : : 4 :
    S  : : : : : 1 : 1 : : :
    T  : 2 : 1 : : : : : : :
    V  : : 3 : 2 : 1 : 3 : :
    W  : : : : : : : : : : :
    Y  : : 5 6 : : : : 4 : 4
  . 
               bits      6.5            
                      5.8            
                      5.2            
                      4.5            
      Information   3.9              
      content   3.2               
    (28.3 bits) 2.6                   
                      1.9                       
                      1.3                       
                      0.6                       
                      0.0    
  . 
    Multilevel                 D      A      Y      Y      A      G      A      G      Y      R      F   
    consensus                 K    Q    V    H    L    R    L      V    D    Y  
    sequence                    T        V            K    
                                                
                                                

  . 
  NAME       START   P-VALUE           SITES   
  rMotifGen_RANDOM_2    196  1.63e-10 
    NNGGLYEPGI     K      T      F      Y      L      G      A      G      Y      R      Y       PAPDLTGMDE 
  rMotifGen_RANDOM_8    446  1.78e-10 
    VKAADREARI     K      T      Y      H      V      G      L      G      F      D      Y       EVDHALAKSV 
  rMotifGen_RANDOM_9    398  3.69e-10 
    TIDSTIFLEL     D      A      Y      Y      A      R      A      G      V      D      F       VAKVHVQGHV 
  rMotifGen_RANDOM_5    171  3.69e-10 
    SYNTVSRVVV     D      Q      Y      H      A      S      A      G      Y      K      F       TATSMAHLPI 
  rMotifGen_RANDOM_3    94  3.69e-10 
    IIPENQDGRA     K      A      V      Y      A      G      L      G      V      K      F       IFHVKREVVV 
  rMotifGen_RANDOM_10    142  9.29e-10 
    TLLPGPAEPS     P      N      Y      H      A      G      L      G      L      R      Y       LVSDNCYKWK 
  rMotifGen_RANDOM_7    81  1.28e-09 
    TVAMPGLQGF     N      A      V      Y      I      G      L      G      V      R      Y       LMKKIMDNTS 
  rMotifGen_RANDOM_4    240  4.62e-09 
    ERAHFGTEGG     D      Q      V      T      L      G      A      G      M      D      F       AELALAPTSE 
  rMotifGen_RANDOM_6    292  1.17e-08 
    GTFKKVATDE     R      P      Y      Y      F      G      M      S      Y      N      F       ESKMGVILIY 
  rMotifGen_RANDOM_1    216  3.43e-08 
    TTAASTIANE     D      A      H      Y      V      R      V      N      Y      R      F       GRVVGKGGEP 
 
    
      	Motif 4 block diagrams  
    
 
  Name Lowest p-value    Motifs
 
     rMotifGen_RANDOM_2
  1.6e-10
    
    
   4
    
   
  
 
     rMotifGen_RANDOM_8
  1.8e-10
    
    
   4
    
   
  
 
     rMotifGen_RANDOM_9
  3.7e-10
    
    
   4
    
   
  
 
     rMotifGen_RANDOM_5
  3.7e-10
    
    
   4
    
   
  
 
     rMotifGen_RANDOM_3
  3.7e-10
    
    
   4
    
   
  
 
     rMotifGen_RANDOM_10
  9.3e-10
    
    
   4
    
   
  
 
     rMotifGen_RANDOM_7
  1.3e-09
    
    
   4
    
   
  
 
     rMotifGen_RANDOM_4
  4.6e-09
    
    
   4
    
   
  
 
     rMotifGen_RANDOM_6
  1.2e-08
    
    
   4
    
   
  
 
     rMotifGen_RANDOM_1
  3.4e-08
    
    
   4
    
   
  
  SCALE
     
     | 
     | 
     | 
     | 
     | 
     | 
     | 
     | 
     | 
     | 
     | 
     | 
     | 
     | 
     | 
     | 
     | 
     | 
     | 
     | 
    1 
     25 
     50 
     75 
     100 
     125 
     150 
     175 
     200 
     225 
     250 
     275 
     300 
     325 
     350 
     375 
     400 
     425 
     450 
     475 
   
 

    
      	Motif 4 in BLOCKS format  
    
 
 
 
 
  
 to  BLOCKS multiple alignment processor.  
    
      	Motif 4 position-specific scoring matrix  
    
 
 

    
      	Motif 4 position-specific probability matrix  
    
 
 

    
      	Motif 4 regular expression  
    
[DK][AQT][YV][YH][ALV][GR][AL]G[YV][RDK][FY]

 


Time 16.41 secs.

 

    
   
     P  
     N        MOTIF  5       width = 14         sites =   2        llr = 78        E-value = 6.3e+000 
    
 
    Simplified  A  : : : : : : : : : : 5 : : :
    pos.-specific  C  : : : : : : : : : : : : a :
    probability  D  : 5 : : : : 5 : 5 : : : : :
    matrix  E  : : : : : : : : : : : : : :
    F  : : : : : : : : : : : a : :
    G  : : : : : : : : : : : : : :
    H  : : : : : : : : : : : : : :
    I  : : 5 : : : : : : : : : : :
    K  : 5 : 5 : 5 : : : : 5 : : a
    L  : : : : : 5 : : : : : : : :
    M  : : : : : : : : : : : : : :
    N  : : : : : : : : : : : : : :
    P  : : : : : : : : : : : : : :
    Q  : : : : : : 5 : : 5 : : : :
    R  : : : : : : : : : : : : : :
    S  : : : 5 : : : a : 5 : : : :
    T  : : : : : : : : : : : : : :
    V  : : : : : : : : : : : : : :
    W  : : 5 : a : : : : : : : : :
    Y  a : : : : : : : 5 : : : : :
  . 
               bits      6.5                
                      5.8                 
                      5.2                 
                      4.5                    
      Information   3.9                      
      content   3.2                           
    (56.0 bits) 2.6                             
                      1.9                             
                      1.3                             
                      0.6                             
                      0.0    
  . 
    Multilevel                 Y      D      I      K      W      K      D      S      D      Q      A      F      C      K   
    consensus                   K    W    S      L    Q      Y    S    K        
    sequence                                            
                                                      
                                                      

  . 
  NAME       START   P-VALUE           SITES   
  rMotifGen_RANDOM_5    66  4.85e-18 
    RDRYYLESKY     Y      D      W      S      W      L      Q      S      D      Q      A      F      C      K       LLRKNLSLKD 
  rMotifGen_RANDOM_4    44  1.38e-16 
    AVSRVVLIGK     Y      K      I      K      W      K      D      S      Y      S      K      F      C      K       LKALKIGRAP 
 
    
      	Motif 5 block diagrams  
    
 
  Name Lowest p-value    Motifs
 
     rMotifGen_RANDOM_5
  4.8e-18
    
    
   5
    
   
  
 
     rMotifGen_RANDOM_4
  1.4e-16
    
    
   5
    
   
  
  SCALE
     
     | 
     | 
     | 
     | 
     | 
     | 
     | 
     | 
     | 
     | 
     | 
     | 
     | 
     | 
     | 
     | 
     | 
     | 
     | 
     | 
    1 
     25 
     50 
     75 
     100 
     125 
     150 
     175 
     200 
     225 
     250 
     275 
     300 
     325 
     350 
     375 
     400 
     425 
     450 
     475 
   
 

    
      	Motif 5 in BLOCKS format  
    
 
 
 
 
  
 to  BLOCKS multiple alignment processor.  
    
      	Motif 5 position-specific scoring matrix  
    
 
 

    
      	Motif 5 position-specific probability matrix  
    
 
 

    
      	Motif 5 regular expression  
    
Y[DK][IW][KS]W[KL][DQ]S[DY][QS][AK]FCK

 


Time 20.40 secs.

 

    
   
     P  
     N        MOTIF  6       width = 27         sites =   2        llr = 139        E-value = 1.3e+001 
    
 
    Simplified  A  : : : : : : : : : : : : : 5 : : : : : : : : : : : : :
    pos.-specific  C  : : : : : : : : : : : : : : : : : : : : : : : 5 : : :
    probability  D  : : : : : : : 5 : 5 : : : : : : 5 : : : 5 : : : : : :
    matrix  E  : : : : : : : : : : : : : 5 : : : : : : : : : : : : :
    F  : : : : : : : : a : : : : : : : : : : : : : : : : : :
    G  : : : : : : : : : : 5 : : : : : : : : : : : : : : : :
    H  : : : : : : : : : : : : : : : : : : : : : : : : : : :
    I  : : : : : : : : : : : : : : : : : : : : 5 : : : 5 a :
    K  : : : : 5 : : 5 : : : 5 : : : : : 5 : : : : : : : : :
    L  : 5 : : : : : : : : : : a : : : 5 : a : : a : : 5 : :
    M  : : : : : : : : : : : : : : : : : : : a : : 5 5 : : :
    N  : : 5 : : 5 : : : : : : : : a 5 : : : : : : : : : : :
    P  : 5 : a 5 : : : : : : : : : : : : : : : : : 5 : : : 5
    Q  : : : : : : : : : 5 : : : : : : : : : : : : : : : : :
    R  : : : : : : 5 : : : : : : : : : : : : : : : : : : : :
    S  : : : : : : : : : : 5 : : : : : : : : : : : : : : : :
    T  : : : : : 5 : : : : : 5 : : : 5 : 5 : : : : : : : : :
    V  : : : : : : : : : : : : : : : : : : : : : : : : : : :
    W  : : : : : : 5 : : : : : : : : : : : : : : : : : : : 5
    Y  a : 5 : : : : : : : : : : : : : : : : : : : : : : : :
  . 
               bits      6.5                            
                      5.8                             
                      5.2                             
                      4.5                                    
      Information   3.9                                       
      content   3.2                                                   
    (100.1 bits) 2.6                                                       
                      1.9                                                       
                      1.3                                                       
                      0.6                                                       
                      0.0    
  . 
    Multilevel                 Y      L      N      P      K      N      R      D      F      D      G      K      L      A      N      N      D      K      L      M      D      L      M      C      I      I      P   
    consensus                   P    Y      P    T    W    K      Q    S    T      E      T    L    T        I      P    M    L      W  
    sequence                                                                      
                                                                                
                                                                                

  . 
  NAME       START   P-VALUE           SITES   
  rMotifGen_RANDOM_5    472  8.58e-30 
    IALGTKHAFA     Y      P      N      P      K      T      W      K      F      D      G      T      L      A      N      N      L      K      L      M      I      L      M      C      I      I      P       GR 
  rMotifGen_RANDOM_7    287  3.41e-29 
    EFMLEAFKAV     Y      L      Y      P      P      N      R      D      F      Q      S      K      L      E      N      T      D      T      L      M      D      L      P      M      L      I      W       RQEDSDRLIK 
 
    
      	Motif 6 block diagrams  
    
 
  Name Lowest p-value    Motifs
 
     rMotifGen_RANDOM_5
  8.6e-30
    
    
   6
    
   
  
 
     rMotifGen_RANDOM_7
  3.4e-29
    
    
   6
    
   
  
  SCALE
     
     | 
     | 
     | 
     | 
     | 
     | 
     | 
     | 
     | 
     | 
     | 
     | 
     | 
     | 
     | 
     | 
     | 
     | 
     | 
     | 
    1 
     25 
     50 
     75 
     100 
     125 
     150 
     175 
     200 
     225 
     250 
     275 
     300 
     325 
     350 
     375 
     400 
     425 
     450 
     475 
   
 

    
      	Motif 6 in BLOCKS format  
    
 
 
 
 
  
 to  BLOCKS multiple alignment processor.  
    
      	Motif 6 position-specific scoring matrix  
    
 
 

    
      	Motif 6 position-specific probability matrix  
    
 
 

    
      	Motif 6 regular expression  
    
Y[LP][NY]P[KP][NT][RW][DK]F[DQ][GS][KT]L[AE]N[NT][DL][KT]LM[DI]L[MP][CM][IL]I[PW]

 


Time 24.40 secs.

 

      
   
     P  
     N        SUMMARY OF MOTIFS   
    
      
 	Combined block diagrams: non-overlapping sites with p-value      
  Name Combined p-value    Motifs
 
     rMotifGen_RANDOM_1
  9.61e-35
    
    
   3
    
   1
    
   4
    
   
  
 
     rMotifGen_RANDOM_2
  3.98e-49
    
    
   3
    
   4
    
   1
    
   2
    
   
  
 
     rMotifGen_RANDOM_3
  6.30e-37
    
    
   4
    
   1
    
   2
    
   
  
 
     rMotifGen_RANDOM_4
  4.57e-44
    
    
   1
    
   5
    
   4
    
   2
    
   
  
 
     rMotifGen_RANDOM_5
  2.40e-58
    
    
   5
    
   1
    
   4
    
   6
    
   
  
 
     rMotifGen_RANDOM_6
  1.11e-50
    
    
   1
    
   3
    
   4
    
   4
    
   2
    
   
  
 
     rMotifGen_RANDOM_7
  9.86e-58
    
    
   4
    
   6
    
   1
    
   2
    
   
  
 
     rMotifGen_RANDOM_8
  6.38e-35
    
    
   2
    
   4
    
   1
    
   4
    
   
  
 
     rMotifGen_RANDOM_9
  2.60e-45
    
    
   6
    
   1
    
   2
    
   4
    
   3
    
   
  
 
     rMotifGen_RANDOM_10
  1.73e-49
    
    
   2
    
   3
    
   4
    
   1
    
   
  
  SCALE
     
     | 
     | 
     | 
     | 
     | 
     | 
     | 
     | 
     | 
     | 
     | 
     | 
     | 
     | 
     | 
     | 
     | 
     | 
     | 
     | 
     | 
    1 
     25 
     50 
     75 
     100 
     125 
     150 
     175 
     200 
     225 
     250 
     275 
     300 
     325 
     350 
     375 
     400 
     425 
     450 
     475 
     500 
   
 
 
     Motif summary in machine readable format. 

    
      Stopped   because nmotifs = 6 reached.
    
 

CPU: compute-0-1.local

 

 

 
   
EXPLANATION OF MEME RESULTS
   

 The MEME results consist of: 

 
   
	The   version  
        of MEME and the date it was released.
   
	The   reference  
        to cite if you use MEME in your research.
   
	A description of the
          sequences  
        you submitted (the "training set") showing the name,
	"weight" and length of each sequence.
   
	The   command line summary
           detailing the parameters with which you ran MEME.
   
	Information on each of the
          motifs   MEME discovered, including:
 
     A  
	summary line 
        showing the width, number of occurrences, log likelihood ratio
	and statistical significance of the motif.
     A  
        simplified position-specific probability matrix .
     A  
	diagram 
        showing the degree of conservation at each motif position.
     A  
        multilevel consensus sequence  
	showing the most conserved letter(s) at each motif position.
     The  
	occurrences of the motif 
	sorted by  p -value and aligned with each other.
 	 
	Block diagrams  
	of the occurrences of the motif within each sequence in the training 
	set.
     The motif in 
	 
	BLOCKS or FASTA format .
     A  
        position-specific scoring matrix (PSSM)  
	for use by the
         MAST  database search program.
     The  
        position specific probability matrix (PSPM)  
	describing the motif.
     A  
        regular expression  describing the motif.
 

   
	A   summary of motifs  
        showing an optimized (non-overlapping) 
         tiling  of all of the motifs onto
        each of the sequences in the training set.
   
	The reason why MEME  stopped 
	and the name of the CPU on which it ran.
   
	This  explanation  of how to interpret MEME results.
 

 
  MOTIFS  

 
For each motif that it discovers in the training set,
MEME prints the following information:
 
 
 
    Summary Line  

This line gives the width (`width'), 
number of occurrences in the training set (`sites'), log likelihood
ratio (`llr') and  E -value of the motif.
Each motif describes a pattern of a fixed width--no gaps are allowed in
MEME motifs.
MEME numbers the motifs consecutively from one as it finds them. 
MEME usually finds the most statistically significant (low  E -value) 
motifs first.
The statistical significance of a motif is based on its log likelihood ratio,
its width and number of occurrences, the background letter frequencies
(given in the  command line summary ), and 
the size of the training set.  The  E -value is an
estimate of the expected number of motifs with the given log likelihood
ratio (or higher), and with the same width and number of occurrences,
that one would find in a similarly sized set of random sequences. 
(In random sequences each position is independent with letters chosen
according to the background letter frequencies.)  The log likelihood
ratio is the logarithm of the ratio of the probability of the occurrences 
of the motif given the motif model (likelihood given the motif) 
versus their probability given the background model (likelihood given the
null model).  (Normally the background model is a 0-order Markov model
using the background letter frequencies, but higher order Markov models
may be specified via the  -bfile  option to MEME.)

Clicking on the  buttons  to the left of the motif summary line 
takes you to the previous motif (P) or next motif (N).

 
    Simplified 
  Position-Specific Probability Matrix  

MEME motifs are represented by position-specific probability matrices 
that specify the probability of each possible letter appearing at each
possible position in an occurrence of the motif.  In order to make it easier 
to see which letters are most likely in each of the columns of the
motif, the simplified motif shows the letter probabilities multiplied by 10 
rounded to the nearest integer ("a" means 10).  Zeros are replaced by ":" 
(the colon) for readability.

 
    Information Content Diagram  

The information content diagram provides 
an idea of which positions in the motif are most highly conserved.
Each column (position) in a motif can be characterized by the amount of
information it contains (measured in bits).  Highly conserved positions
in the motif have high information; positions where all letters are equally
likely have low information.  (The information content is relative to
the background letter frequencies which are given in the 
 command line summary  section.)
The diagram is printed so that each column lines up with the same column in 
the simplified position-specific probability matrix above it.

Columns in the information content diagram are colored according to the
majority category of the letters occurring in that column of the alignment.
If no letter category has frequency above 0.5, the column in the diagram
is colored black.  For DNA sequences, the letter categories contain one letter
each.  For proteins,  the categories are based on the biochemical properties
of the various amino acids.  The categories and their colors are:
 
 
   
       NUCLEIC ACIDS   COLOR   
      
       A  
        RED   
      
        C  
         BLUE   
      
       G  
        ORANGE   
      
       T  
        GREEN   
   
   
   
       AMINO ACIDS   COLOR  
	  PROPERTIES   
       A, C, F, I, L, V, W and M 
        BLUE  
       Most hydrophobic[Kyte and Doolittle, 1982] 
     
     
       NQST 
        GREEN  
       Polar, non-charged, non-aliphatic residues 
     
     
       DE 
        MAGENTA  
       Acidic 
     
     
       KR 
        RED  
       Positively charged 
     
     
       H 
        PINK    
     
       G 
        ORANGE    
     
       P 
        YELLOW    
     
       Y 
        TURQUOISE    
   
 
 
J. Kyte and R. Doolittle, 1982.
"A Simple Method for Displaying the Hydropathic Character of a Protein",
J. Mol Biol. 157, 105-132.

 
Summing the information content for each position in the motif gives
the total information content of the motif (shown in parentheses to the
left of the diagram).  The total information content is approximately
equal to the log likelihood ratio divided by the number of occurrences times
ln(2).
The total information content gives a measure of
the usefulness of the motif for database searches.
For a motif to be useful for database searches, it must as a rule contain at 
least log_2(N) bits of information 
where N is the number of sequences in the database being searched.  
For example, to effectively search a database containing 100,000 sequences
for occurrences of a  single  motif, the motif should have an IC of at
least 16.6 bits.  Motifs with lower information content are still useful when a 
family of sequences shares more than one motif since they can be combined
in  multiple  motif searches (using MAST).

 
    Multilevel 
  Consensus Sequence  

The multilevel consensus sequence corresponding to the motif is an aid in 
remembering and understanding the motif.   It is calculated from the motif 
position-specific probability matrix as follows.  
Separately for each column of the motif, 
the letters in the alphabet are sorted in decreasing order by the probability 
with which they are expected to occur in that position of motif occurrences.  
The sorted letters are then printed vertically with the most probable letter 
on top.  Only letters with probabilities of 0.2 or higher at that position in 
the motif are printed.  As an example, the multilevel consensus sequence of 
motif 1 in the sample output is:
 
 Multilevel   TTATGTGAACGACGTCACACT 
 consensus   AA  T A G A GA     AA
 sequence            T C TT     T
 
This multilevel consensus sequence says several things about the motif.
First, the most likely form of the motif
can be read from the top line as 
 TTATGTGAACGACGTCACACT .
Second, that only letter  A  has probability more than 0.2 in
position 3 of the motif, both  T  and  A  have probability
greater than 0.2 in position 1, etc.
Third, a  rough approximation  of the motif can be made by converting the
multilevel consensus sequence into a 
 regular expression  for the motif.
  
 [TA][TA]AT[GT][T][GA]A[AGT]C[GAC]A[CGT][GAT]TCACA[CAT][TA] 
 

 
    Occurrences of the Motif  

MEME displays the occurrences (sites) of the motif in the training set.  
The sites are shown aligned with each other, and the ten sequence
positions preceding and following each site are also shown.
Each site is identified by the name of the sequence where it occurs,
the strand (if both strands of DNA sequences are being used), and the
position in the sequence where the site begins.  When the DNA strand
is specified, `+' means the sequence in the training set,
and `-' means the reverse complement of the training set sequence.
(For `-' strands, the `start' position is actually the position on the
 positive  strand where the site ends.)
The sites are listed in order of increasing statistical significance
( p -value).  The  p -value of a site is computed from the the match 
score of the site with the  position specific scoring matrix  
for the motif.  The  p -value gives the probability of a random string
(generated from the background letter frequencies) having the same match
score or higher.  (This is referred to as the  position  p -value 
by the MAST algorithm.)

 
    
Block Diagrams of Motif Occurrences 
 
The occurrences of the motif in the training set sequences are 
shown with MAST-style block diagrams.  One diagram is printed for each
sequence showing all the occurrences of the motif in that sequence.
The sequences are sorted by the  lowest   p -value among all 
occurrences of the motif in a given sequence.
(The  p -value of an occurrence is the probability of a single
random subsequence the length of the motif,
generated according to the 0-order background model, having a score
at least as high as the score of the occurrence.)
When the DNA strand is specified, `+' means the motif appears from left to
right on the sequence, and `-' means the motif appears from right to left 
on the complementary strand.
A sequence position scale is shown at the end of each table of block
diagrams.  Very long sequences are shown with thick lines connecting the
motifs and are  not  drawn to scale.

 
    
Motif in BLOCKS format or FASTA format 
 

For use with 
 BLOCKS tools , 
MEME prints the occurrences of the motif in BLOCKS format. 
 
You can convert these blocks to 
PSSMs (position-specific scoring matrices), LOGOS (color representations
of the motifs), phylogeny trees and search them against a database of other
blocks by pasting everything from the "BL" line to the "//" line (inclusive)
into the  
 
Multiple Alignment Processor.  
If you include the  -print_fasta  switch on the command line, MEME prints
the motif sites in FASTA format instead of BLOCKS format.

 
    Position-Specific Scoring Matrix  

The position-specific scoring matrix corresponding to the motif is printed
for use by database search programs such as MAST.  This matrix is a
log-odds matrix calculated
by taking 100 times the log (base 2) of the ratio  p/f  at each position in
the motif where  p  is the probability of a particular letter at that
position in the motif, and  f  is the background frequency of the
letter (given in the  command line summary  section.)
This is the same matrix that is used above in computing the  p -values
of the occurrences of the motif in the  Occurrences of the
Motif  and  Block Diagrams of Motif Occurrences 
sections.  
The scoring matrix is printed "sideways"--columns
correspond to the letters in the alphabet (in the same order as shown in
the simplified motif) and rows corresponding to the positions of the motif,
position one first.  The scoring matrix is preceded by a line starting with
"log-odds matrix:" and containing the length of the alphabet, width
of the motif, number of characters in the training set, the scoring 
threshold (obsolete) and the motif  E -value.
 
 Note:  The probability  p  used to compute the PSSM
is  not  exactly the same as the corresponding value in the 
Position Specific Probability Matrix (PSPM).  
The values of  p  used to compute the PSSM take
into account the motif prior, whereas the values in the PSPM are just
the  observed  frequencies of letters in the motif sites.

 
    
Position-Specific Probability Matrix  

The motif itself is a position-specific probability matrix giving,
for each position in the pattern, the observed frequency 
("probability") of each possible letter.  
The probability matrix is printed "sideways"--columns
correspond to the letters in the alphabet (in the same order as shown in
the simplified motif) and rows corresponding to the positions of the motif,
position one first.
The motif is preceded by a line starting with
"letter-probability matrix:" and containing the length of the alphabet, width
of the motif, number of occurrences of the motif, and the  E -value of the
motif.
 
 Note:  Earlier versions
of MEME gave the posterior probabilities--the probability after applying
a prior on letter frequencies--rather than the observed frequencies.
These versions of MEME also gave the number of  possible 
positions for the motif rather than the actual number of occurrences.
The output from these earlier versions of MEME can be distinguished
by "n=" rather than "nsites=" in the line preceding the matrix.

 
    
Regular Expression  
This is the  multilevel consensus  expressed as
a regular expression for convenience.  Regular expressions can
be used for searching for against sequences (using, for example,
 PatMatch )  
but the search accuracy will usually be better with the PSSM (using,
for example
 MAST .)
MEME regular expressions are interpreted as follows: 
single letters match that letter; groups of letters in square brackets 
match any of the letters in the group.

 
    
Motif Summary Tiling  
The motif summary tiling is done using the same algorithm as used
by  MAST .
The motif occurrences shown in the motif summary
 may not be exactly the same as those reported in each motif section 
because only motifs with a position  p -value of 0.0001 that
don't overlap other, more significant motif occurrences are shown.
The format of the machine readable motif-summary is:
 
[sequence_name combined_ p -value number_of_motif_occurrences [motif_number start_of_motif position_ p -value]+]+
 
See the documentation for 
 MAST output  for the definition of position and
combined  p -values.

 
   
     Go to top    
 
 
 
